# Sepsis-induced coagulopathy: recent insights on the role and clinical application of neutrophil extracellular trap formation

**DOI:** 10.1038/s41598-025-31771-y

**Published:** 2025-12-31

**Authors:** Shams ElDoha Galal ElDin Zaiema, Nahela Ahmed Shalaby, Toka Hassaan Mohamed, Aya Ali Mahmoud Bayoumy, Raghda El Sayed Abdel Monem Galal

**Affiliations:** 1 Department of Clinical Pathology, Ain Shams University, Cairo, Egypt; 2 Department of Anaesthesia and ICU, Ain Shams University, Cairo, Egypt

**Keywords:** Sepsis-induced NETosis, Myeloperoxidase (MPO), Histones (HIST), Immunothrombosis, Response, Short-term mortality, Sepsis management in critical care units, Biomarkers, Diseases, Immunology, Medical research

## Abstract

Sepsis is characterized by life-threatening organ dysfunction caused by an uncontrolled immune response to infection. Neutrophils play a vital role in this process, which can lead to immunothrombosis and disseminated intravascular coagulation (DIC) via the formation of neutrophil extracellular traps (NETs). This study aimed to validate the impact of NETs biomarkers in evaluating their potential as diagnostic, prognostic, and therapeutic indicators in critical care for patients with sepsis. We conducted a case-control study with a 7-day follow-up to assess mortality in 138 sepsis patients, focusing specifically on the occurrence of DIC. Additionally, 80 healthy volunteers, matched by age and sex, served as controls. Our findings reveal a strong connection between histones in sepsis and both the initial inflammatory response and sepsis-related coagulopathy/DIC. Furthermore, we found that myeloperoxidase can effectively predict short-term mortality among sepsis patients, regardless of their DIC status. This study highlights a concerning simultaneous increase in myeloperoxidase and histones (thresholds of > 84.9 ng/ml and > 126.4 ng/ml, respectively), which may serve as vital indicators indicating the urgent need for NETs inhibitors in sepsis treatment. Applying this approach, we anticipate a significant reduction in thrombotic events and mortality, thereby enhancing patient care and outcomes in the management of critical sepsis.

## Introduction

Sepsis is a complex syndrome triggered by infections that can result in organ dysfunction, as indicated by an elevated Sequential Organ Failure Assessment (SOFA) score^1^. A key component of sepsis is sepsis-induced coagulopathy (SIC), which arises from an uncontrolled acquired immunoinflammatory response and the stimulation of coagulation mechanisms. Overt disseminated intravascular coagulation (DIC) represents an advanced stage of this condition, characterized by extensive coagulation and organ failure^2,3^.

Recent studies have highlighted the crucial roles of immune cells, including neutrophils and monocytes, in promoting blood clot formation within blood vessels, a phenomenon known as immunothrombosis^4^. When endothelial cells encounter pathogens, they increase their adhesion capacity, enabling the attachment of leukocytes. Activated monocytes release tissue factor, which initiates coagulation and fibrin fiber formation, further drawing in and activating leukocytes via the integrin αMβ2 (Mac-1)^4–6^ Furthermore, neutrophils play a crucial role by linking inflammation and coagulation through the formation of neutrophil extracellular traps (NETs) during a specific mode of cell death known as NETosis. NETs can profoundly affect coagulopathy by interacting with various biological systems, obstructing fibrinolysis, and disrupting natural anticoagulant mechanisms, thereby contributing to thrombocytopenia^5,6^.

Myeloperoxidase (MPO) plays a vital role in the neutrophil extracellular trap framework^7^. This enzyme, which is abundant in neutrophils, is crucial for the inflammatory response and the production of reactive oxygen species (ROS), which can cause vascular inflammation and damage, activate clotting factors and platelets, and ultimately lead to thrombosis and coagulopathy^4–6^. Histones (HIST) are also key in NETosis, exhibiting procoagulant properties while opposing natural anticoagulant mechanisms such as thrombomodulin-mediated protein C activation, and they also reduce the effectiveness of Antithrombin (AT)^4,8–10^.

Research consistently shows that excessive NETs formation can cause significant inflammation, organ damage, disruption of the coagulation system, impaired platelet function, and endothelial cell injury^11^. These issues can lead to serious outcomes, including bleeding, DIC, or even death^11–13^. Therefore, understanding how NET-mediated coagulopathy occurs in sepsis is crucial for developing targeted treatments that can be applied early in SIC cases, especially when coagulation changes are reversible^12,13^. Fortunately, ongoing research has identified various NETosis inhibitors, such as toll-like receptor antagonists and reactive oxygen species scavengers, which show promise in preventing excessive NETs formation. These strategies aim to balance the beneficial role of NETs activation in host defense with its potential harmful effects.

This study investigates the impact of NETs biomarkers, specifically MPO and HIST, on the mechanisms underlying sepsis and sepsis-induced coagulopathy. It also aims to evaluate their relationships with various clinical and laboratory indicators while exploring their potential as diagnostic, prognostic, and therapeutic markers in critical care settings.

## Patients and methods

### The study protocol and setting

We used a case-control study design with a 7-day follow-up for the cases to assess mortality, involving 218 individuals—138 newly diagnosed sepsis patients and 80 healthy controls matched by age and sex—recruited from April 2024 to April 2025. All participants or their guardians (if disoriented or comatose) provided written informed consent. The cohort of sepsis patients (*n* = 138) was sourced from the intensive care units (ICUs) at Ain Shams University Hospital in Cairo, Egypt.

The 80 healthy volunteer subjects, matched for age and sex, were enrolled as a control group. Their blood samples were collected at Ain Shams Hospital labs after routine follow-up investigations, pre-employment screening, or before surgical procedures (as documented in history taking and lab results). The patient-to-control ratio was not 1:1, as the primary purpose of including healthy controls was to establish a normal range or cutoff value for NETs activation in our patients, as no reference values have been set to date. Additionally, the study focuses on sepsis patients and the underlying NETs-related coagulopathy rather than on a case-control comparison. Nonetheless, including a control group was deemed necessary.

Diagnosis of sepsis in patients was made using the Sequential Organ Failure Assessment (SOFA), which confirms inclusion of those with at least two organ failures—covering respiratory, cardiovascular, hepatic, neurological, coagulation, and renal functions. Infection was verified through microbiological testing.

The presence of DIC, as identified by the JAAM-2 DIC score (≥ 3), enabled the classification of sepsis patients into two subgroups: a positive DIC subgroup (*n* = 30) and a negative DIC subgroup (*n* = 108). (DIC Score details demonstrated in Table 1).

Based on the 7-day mortality rates among the sepsis patients, they were further categorized into the 7-day Died group (*n* = 24) and the 7-day Alive group (*n* = 114). All patients were followed up until day 7 to assess mortality outcomes.

Most patients received anticoagulant therapy, primarily heparin, in conjunction with catecholamine (±), and antimicrobial treatment, which was either broad-spectrum or tailored to the results of culture and sensitivity tests. Information regarding each patient’s medical history was gathered from their hospital records.

### Data sources

Medical history data were collected from the patients’ hospital information system (HIS), including Patient identification, medical history, physical examination, surgical records, the patient‘s diagnosis, and treatment. All entries are signed, dated, accessed, and monitored in accordance with ASU Quality Unit and ethical standards.

**Fig. 1:**
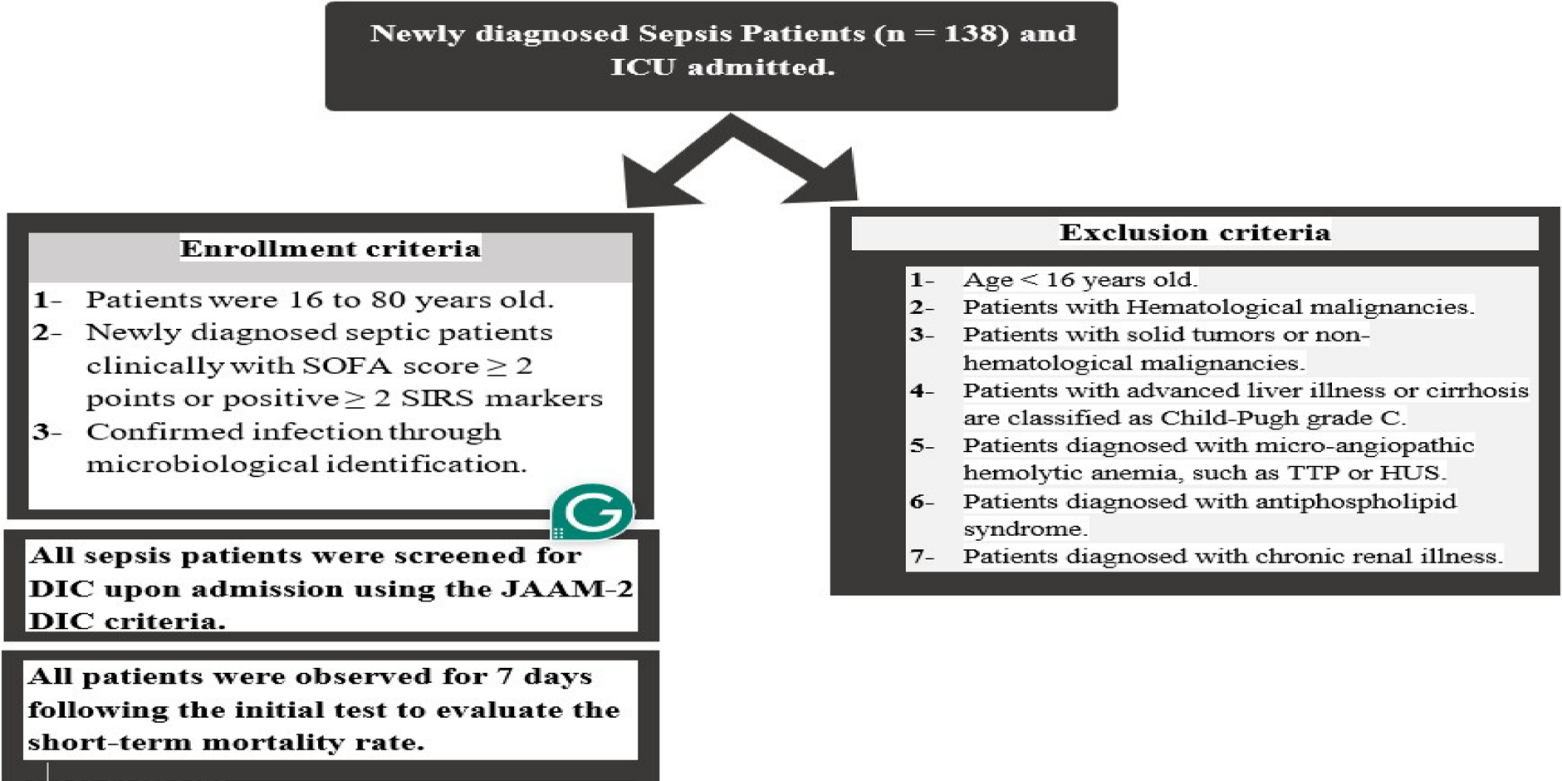
Patient enrollment and exclusion criteria.

Enrollment and Exclusion criteria are demonstrated in the following diagram (Figure 1):

### Methods

#### ASU ethical committee approval code/license

All experimental protocols were approved per the ethical committee regulations of Ain Shams University (ASU).

#### Sampling method

Sets of peripheral venous blood samples were collected from patients upon admission after the sepsis diagnosis was confirmed by SOFA score, using EDTA, sodium citrate (3.2%), and anticoagulant-free tubes. The first 7–10 mL collected upon admission were cultured in a BACT/Alert PF Plus blood culture bottle.

#### Clinical and laboratory procedures for participants

1. **Clinical assessment**: Evaluated for SOFA scores.
2. **Laboratory evaluation**: This included an arterial blood gas test, liver and kidney function, procalcitonin, CRP, ESR, microbiological evaluation, a complete blood count (CBC) with a focus on WBCs, the neutrophil-to-lymphocyte ratio (NLR, calculated as the absolute neutrophil count divided by the absolute lymphocyte count), and platelet count. Coagulation Profile: (PT, INR, PTT, FDPs, and D-dimer) and assessment of PAI-1 (Bioassay Technology Laboratory, Cat. No. E1159Hu, Shanghai, China) using a sandwich ELISA kit.
3. Markers of NETosis formation were assessed using the sandwich ELISA KIT, Human Myeloperoxidase, MPO (Bioassay Technology Laboratory, E0880Hu, Shanghai, China), and Human Citrullinated Histone H3, **Cit-HIST-H3** (MyBioSource, Cat. No MBS1609017, sunny Southern California, San Diego (USA)). Non-fasting serum samples were separated into labelled aliquots, collected, and stored at -20 °C. Assays were done according to the manufacturer’s instructions.
4. **DIC scores calculation**: The JAAM-2 DIC criteria were used to assess DIC. (DIC Score details demonstrated in Table 1).

**Table 1:** DIC scores parameters and calculation.

Parameter	JAAM-2 DIC	Points
Platelet count	< 80 × 10^9^/L or > 50% decrease /24 h	3 Points
	≥ 80 - <120 × 10^9^/L or 30–50% decrease /24 h	1 Point
Prothrombin time [PT]	≥ 1.2s	1 Point
FDP/D-dimer	≥ 25 µg/ml	3 Points
	≥ 10 - <25 µg/ml	1 Point
Required point for the criteria positivity	≥ 3	

### Administrative and ethical design

According to the regulations of Ain Shams University’s ethics committee, each participant provided informed consent to participate in the study prior to enrollment. Confidentiality and privacy were maintained throughout the entire study. All methods and procedures were conducted in accordance with the guidelines and policies of Ain Shams University’s ethical committee (ASU).

#### Statistical methods

Statistical analysis was performed using Stata© software version 17 (StataCorp, 2021). College Station, TX: StataCorp LLC. Numerical variables are presented as Mean ± SD for parametric data and the Median and interquartile range (IQR) for non-parametric data. Categorical variables are presented as percentages of the total. Sensitivity and specificity were calculated using receiver operating characteristic (ROC) curves. For qualitative data, TP and TN, as well as PPV and NPV, were used. A student t-test was used to assess mean differences, while the Mann-Whitney U test was used to evaluate non-parametric differences. The chi-square test was used to examine relationships between qualitative variables. P values < 0.05 are considered statistically significant.

## Results

### Demographic data and comparative studies between the control and sepsis patient groups regarding MPO, CIT-HIST-H3 levels, and other laboratory investigations Table (2)

**Table 2:** Demographic data and comparative studies between the control and sepsis patient groups regarding MPO, CIT-HIST-H3 levels, and other laboratory investigations.

Parameters		Control group	Sepsis patient group	*P*-value
		No. = 80	No. = 138	
Age	Mean ± SD	54.8 ± 18.34	53.93 ± 18.45	0.736
	Range	16–89	16–89	
Sex	Male	32 (40.0%)	50 (36.2%)	0.759*
	Female	48 (60.0%)	88 (63.8%)	
HB g/dL	Mean ± SD	13.12 ± 1.23	9.81 ± 1.81	0.000•
	Range	11.1–15.8	5.1–17	
WBCs (x10^3^/ul)	Mean ± SD	7.74 ± 1.58	13.14 ± 4.94	0.000•
	Range	4.4–10.5	3.4–32.8	
N/L ratio	Median (IQR)	2.19(1.19–3.09)	8.7(6.19–14.47)	0.000≠
	Range	0.68–8.9	0.77–115	
PLTs (x10^3^/ul)	Median (IQR)	264(230–309)	124(66–184)	0.000≠
	Range	181–344	2–602	
PT (sec)	Mean ± SD	13.64 ± 1.66	20.16 ± 9.56	0.017•
	Range	10.6–16.6	12.9–70.5	
INR	Mean ± SD	1.03 ± 0.11	1.84 ± 0.93	0.003•
	Range	0.87–1.25	1.13–5.84	
PTT (sec)	Mean ± SD	33.74 ± 6.46	53.3 ± 29.18	0.050•
	Range	20.2–40.7	23.9–140	
CRP (< 5 mg/L)	Median (IQR)	5 (3.5–9)	103 (43.5–203)	0.000≠
	Range	0.3–22.1	15–396	
FDPs (< 5 Ug/ml)	Median (IQR)	–	24.3 (12–40)	–
	Range	–	3–170	
D dimer (0-0.55 Ug/ml)	Median (IQR)	–	2.29 (1.7–5)	–
	Range	–	0.98–7	
Fibrinogen (2–4 g/L)	Median (IQR)	–	3.3 (2–5.1)	–
	Range	–	0.5–9	
PCT (up to 0.05 ng/ml)	Median (IQR)	–	12.3 (2–45)	–
	Range	–	0.05–110	
Total Bilirubin mg/dl	Median (IQR)	–	1.3 (1.25–2.1)	–
	Range	–	0.5–8	
Creatinine mg/dl	Median (IQR)	–	1.5 (1.01–2.01)	–
	Range	–	0.8–5.5	
MPO (ng/ml)	Median (IQR)	2.01 (0.9–2.8)	73.7 (62.1–84.1)	0.000≠
	Range	0.21–3.69	36.4–124.8	
CIT-HIST-H3 (ng/ml)	Median (IQR)	1.38 (0.81–2.19)	106.3 (66.4–193.3)	0.000≠
	Range	0.49–3.62	18.2–376.9	
SOFA score	Median (IQR)	– –	5 (5–8)	–
	Range		3–17	
Overall Mortality rate among the Studied sepsis group		–	49/138 (35.5%)	–
Mortality rate among the Studied sepsis group in the initial 7 days		–	24/138 (17.4%)	–
Catecholamine infusion rate in the sepsis group		–	104/138 (75.4%)	–
Use of mechanical ventilation rate in the sepsis group		–	80/138 (57.9%)	–
Extrarenal replacement therapy (RRT) in the sepsis group		–	5/138 (3.6%)	–

P-value > 0.05: Non-significant; P-value < 0.05: Significant; P-value < 0.01: Highly significant •: Independent t-test; ≠: Mann-Whitney test, *: Chi-square test.

### The microbiological work-up in the studied sepsis population is demonstrated in (Table 3)

**Table 3:** Results of Microbiological work-up in all sepsis patients studied.

Microbiological cultures	Count or proportion	Valid %
Positive blood culture, organism not identified	15/138	20.7%
Positive cultures and identified organisms*	123/138	82.5%
Culture source and infection site**		
Blood	50/138	36.2%
A central venous catheter (CVC)	13/138	9.4%
Urine	20/138	14.49%
Wound pus	28/138	20.2%
Drains (post-surgery)	8/138	5.7%
Sputum/Lung expectoration	13/138	9.4%
Microbe(s) isolated (*n* = 123/138) *		
Klebsiella species	40/123	32.5%
Acinetobacter species	29/123	23.5%
Staphylococcus aureus	18/123	14.6%
Pseudomonas species	14/123	11.4%
E. coli and other Enterobacteriaceae	11/123	8.9%
Candida tropicalis	11/123	8.9%
Other Gram-Negative (Enterobacteriaceae, Proteus, and Providencia)	11/123	8.9%
Streptococcus species	4/123	3.25%
Gram-negative bacteria	105/138	76.0%
Gram-positive bacteria	22/138	15.9%
Fungal infection	11/138	7.97%
Viral infection (Coronaviruses, Enterovirus, and Rhinovirus)	4/138	2.89%

*Positive blood culture, organism not identified, I mean not cultivated by routine basic cultures, which is primarily due to Prior antibiotic treatment.

**Note that some patients showed positive results in multiple cultures and had polymicrobial infections.

### Comparative studies between positive-DIC and negative-DIC sepsis patient subgroups regarding demographic data, MPO, CIT-HIST-H3 levels, and other laboratory investigations (Table 4)

**Table 4:** Comparison of demographic data, laboratory investigations, PAI, SOFA score, 7-day mortality, MPO, and CIT-HIST-H3 between positive-DIC and negative-DIC sepsis patients subgroups.

Sepsis patient group No. = 138		Positive-DIC	Negative-DIC	P-value
		No. = 30	No. = 108	
Age (years)	Mean ± SD	62.93 ± 16.03	50.7 ± 17.96	0.020•
	Range	34–89	16–84	
Sex	Male	8 (26.7%)	42 (38.9%)	0.384*
	Female	22 (73.3%)	66 (61.1%)	
HB g/dL	Mean ± SD	9.24 ± 1.82	9.97 ± 1.79	0.169•
	Range	5.1–11.6	6.8–17	
WBCs (x10^3^/ul)	Mean ± SD	14.27 ± 4.41	12.82 ± 5.07	0.318•
	Range	7–22.2	3.4–32.8	
PNL (x10^3^/ul)	Mean ± SD	13.77 ± 4.23	9.95 ± 4.45	0.004•
	Range	6.3–20.65	2.02–27.54	
LYMP (x10^3^/ul)	Median (IQR)	0.68(0.5–1.11)	1.17(0.8–2.01)	0.006≠
	Range	0.4–1.3	0.14–3.8	
N/L ratio	Median (IQR)	17.84(12.6–26.3)	7.2(5.2–12)	0.000≠
	Range	6.53–36.24	0.77–115	
PLTs (x10^3^/ul)	Median (IQR)	46(30–81)	134.5(100–236)	0.000≠
	Range	2–220	39–602	
PT (sec)	Mean ± SD	27.06 ± 15.51	18.24 ± 6.04	0.001•
	Range	14.2–70.5	12.9–44.8	
INR	Mean ± SD	2.29 ± 1.28	1.71 ± 0.78	0.033•
	Range	1.3–5.84	1.13–4.62	
PTT (sec)	Mean ± SD	65.84 ± 37.76	49.53 ± 25.34	0.057•
	Range	31.3–140	23.9–130	
FDPs (< 5 Ug/ml)	Median (IQR)	45(35.8–58.9)	20.6(10–28.2)	0.000≠
	Range	19–160	3–170	
D dimer (0-0.55 Ug/ml)	Median (IQR)	5(5–6)	2(1.65–3.5)	0.000≠
	Range	2.44–7	0.98–7	
Plasma Fibrinogen*** (2–4 g/L)	Median (IQR)	1.4(0.8–2.9)	4.6(2.95–5.8)	0.000≠
	Range	0.5–6.7	0.8–9	
Procalcitonin (up to 0.05 ng/ml)	Median (IQR)	7.69(0.75–21.7)	6.75(1.38–21.1)	0.952≠
	Range	0.44–51.8	0.05–120	
CRP (< 5 mg/L)	Median (IQR)	101.5(45–110)	112.5(45–224.5)	0.490≠
	Range	24–268	15–396	
PAI (N = 5-20ng/ml)	Mean ± SD	53.81 ± 24.09	40.49 ± 18.47	0.024•
	Range	16.08–87.64	15.39–77.03	
SOFA score	Median (IQR)	5(4–10)	5(4–8)	0.745≠
	Range	3–11	3–17	
7-day mortality	Alive (n = 114)	14 (46.7%)	100 (92.6%)	0.000*
	Died (n = 24)	16 (53.3%)	8 (7.4%)	
MPO (ng/ml)	Median (IQR)	92.5 (56.8–110.9)	72.6 (62.1–80.4)	0.030≠
	Range	48.4–123.3	36.4–124.8	
CIT-HIST-H3 (ng/ml)	Median (IQR)	257.8(141.4–306.3)	85.4 (60.0–135.1)	0.000≠
	Range	53.1–326.0	18.2–376.9	

P-value > 0.05: Non -significant; P-value < 0.05: Significant; P-value < 0.01: Highly significant.

*Chi-square test; •: Independent t-test; ≠: Mann-Whitney test.

*** Plasma fibrinogen levels increase during infection but can decrease due to degradation, as seen in severe coagulopathy, which is common in most of our patients. In some cases, levels are lowered because of degradation or impaired protein synthesis caused by liver disease, especially in the elderly, or in cases of severe sepsis-induced coagulopathy and DIC.

### Comparison between 7-day alive and 7-day died sepsis patients subgroups (regardless of the presence of DIC) (Table 5)

**Table 5:** Comparison between 7-day alive and 7-day died sepsis patients’ subgroups regarding demographic data, laboratory investigations, PAI, SOFA score, MPO, and CIT-HIST-H3.

Sepsis patient group No. = 138		7-day alive	7-day died	P-value
		No. = 114	No. = 24	
Age (years)	Mean ± SD	49.63 ± 17.28	71.08 ± 10.26	0.000•
	Range	16–84	52–89	
Sex	Male	42 (36.8%)	8 (33.3%)	0.818*
	Female	72 (63.2%)	16 (66.7%)	
HB g/dL	Mean ± SD	9.88 ± 1.77	9.49 ± 2.07	0.505•
	Range	6.8–17	5.1–11.6	
WBCs (x10^3^/ul)	Mean ± SD	12.82 ± 4.89	14.67 ± 5.07	0.241•
	Range	5–32.8	3.4–22.2	
PNL (x10^3^/ul)	Mean ± SD	9.98 ± 4.55	14.59 ± 3.02	0.001•
	Range	2.02–27.54	10.98–20.65	
LYMP (x10^3^/ul)	Median (IQR)	1.16 (0.76–2)	0.74 (0.46–1.01)	0.020≠
	Range	0.26–3.8	0.14–2.21	
N/L ratio	Median (IQR)	7.3 (5.88–12)	19.8 (13.96–30.78)	0.000≠
	Range	0.77–36.24	7.07–115	
PLTs (x10^3^/ul)	Median (IQR)	131 (80–236)	68 (37–140)	0.033≠
	Range	2–602	15–220	
PT (sec)	Mean ± SD	18.37 ± 5.77	28.67 ± 17.3	0.000•
	Range	12.9–44.8	14–70.5	
INR	Mean ± SD	1.71 ± 0.77	2.44 ± 1.38	0.012•
	Range	1.13–4.62	1.3–5.84	
PTT (sec)	Mean ± SD	52.89 ± 29.91	55.27 ± 26.49	0.807•
	Range	23.9–140	31.3–130	
FDPs (< 5 Ug/ml)	Median (IQR)	21 (10.1–32)	40.4 (23–52)	0.037≠
	Range	3–170	6.7–160	
D dimer (0-0.55 Ug/ml)	Median (IQR)	2.12 (1.65–3.97)	5 (2.44–5)	0.011≠
	Range	0.98–7	1.73–7	
Fibrinogen (2–4 g/L)	Median (IQR)	4 (2.3–5.75)	2 (0.9–3.5)	0.028≠
	Range	0.7–9	0.5–6.7	
Procalcitonin (Up to 0.05 ng/ml)	Median (IQR)	7.9 (1.39–21.7)	1.6 (0.75–2)	0.125≠
	Range	0.05–120	0.44–9.59	
CRP (< 5 mg/L)	Median (IQR)	110 (45–246)	46.25 (33.75–112)	0.103≠
	Range	15–396	24–120	
PAI (N = 5-20ng/ml)	Mean ± SD	37.23 ± 16.41	72.65 ± 8.35	0.000•
	Range	15.39–71.93	60.56–87.64	
SOFA score	Median (IQR)	5 (4–8)	5 (4–9.5)	0.847≠
	Range	3–16	3–17	
JAAM2 DIC score ≥ 3	Positive	14 (12.3%)	16 (66.7%)	0.000*
	Negative	100 (87.7%)	8 (33.3%)	
MPO (ng/ml)	Median (IQR)	70.4 (58.5–79.0)	109.0 (91.3–117.5)	0.000≠
	Range	36.4–107.8	79.8–124.8	
CIT-HIST-H3 (ng/ml)	Median (IQR)	92.2 (60.0–141.4)	269.9 (194.2–298.1)	0.000≠
	Range	18.2–376.9	81.3–341.3	

P-value > 0.05: Non-significant; P-value < 0.05: Significant; P-value < 0.01: Highly significant.

*: Chi-square test; •: Independent t-test; ≠: Mann-Whitney test.

### Comparison of 7-day alive and 7-day died patients in the Positive-DIC subgroup regarding MPO and CIT-HIST-H3 levels (Table 6)

**Table 6:** Comparison between DIC sepsis patients who survived and died within 7 days.

Positive-DIC sepsis patient No. 30		7-day alive	7-day died	P-value
		No. = 14	No. = 16	
MPO (ng/ml)	Median (IQR)	56.8(50.3–95.8)	110.9 (92.5–123.3)	0.003≠
	Range	48.4–96.8	79.8–124.8	
CIT-HIST-H3 (ng/ml)	Median (IQR)	172.5 (135.0–321.8)	270 (195.2–306.3)	0.298≠
	Range	53.1 – 326.0	92.5– 341.3	

P-value > 0.05: Non-significant; P-value < 0.05: Significant; P-value < 0.01: Highly significant, ≠: Mann-Whitney test.

### Correlation and relationship studies of MPO and CIT-HIST-H3, with other parameters among the studied sepsis patients

**Fig. 2:**
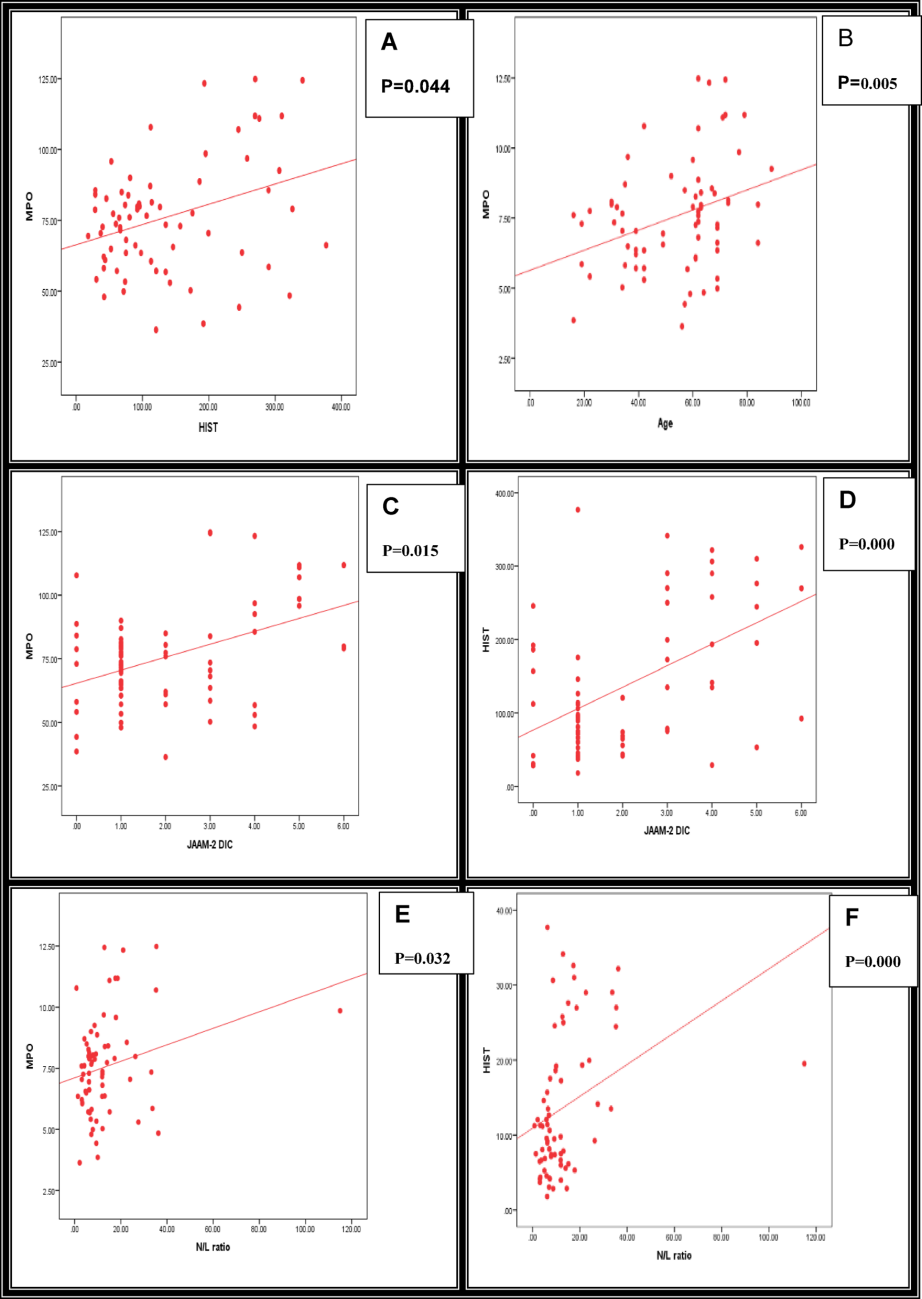

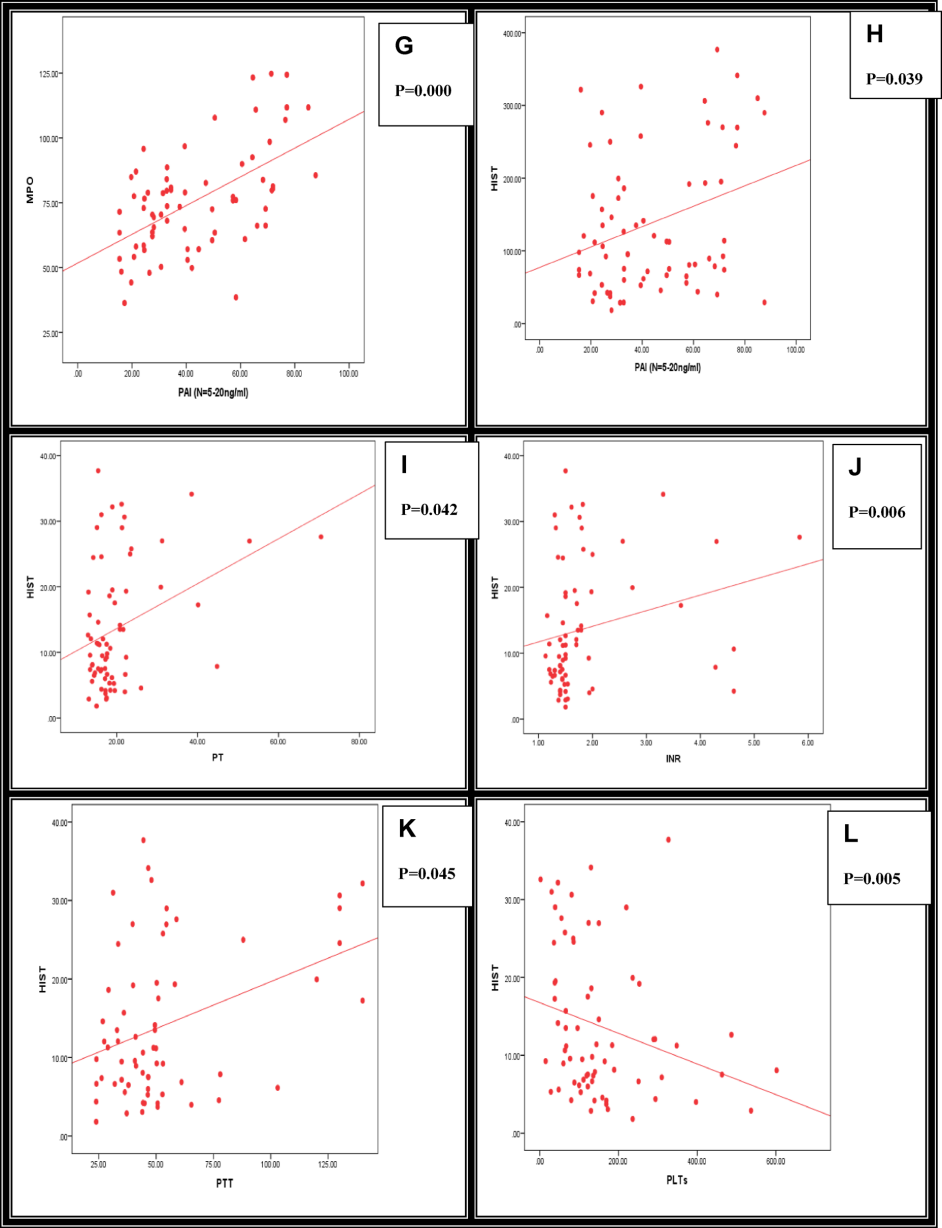
(**A**–**L**): Summarize the Correlation of MPO and CIT-HIST-H3 with other parameters studied among all the groups studied. (**A**) MPO vs. HIST, (**B**) MPO vs. age, (**C**) MPO vs. JAAM-2 DIC ≥ 3, (**D**) HIST vs. JAAM-2 DIC ≥ 3, (**E**) MPO vs. N/L ratio, (**F**) HIST vs. N/L ratio, (**G**) MPO vs. PAI, (**H**) HIST vs. PAI, (**I**) HIST vs. PT, (**J**) HIST vs. INR, (**K**) HIST vs. PTT, (**L**) HIST vs. PLTs. P-value < 0.05: Significant; P-value < 0.01: Highly significant. Spearman correlation coefficient test.

- Relationship studies of MPO and CIT-HIST-H3, with other parameters among the studied sepsis patients, are shown in Table 7.
- Correlation Studies of MPO and CIT-HIST-H3, with other parameters among the studied sepsis patients, are shown in Table 8 and Fig. 2 (A–L).

**Table 7:** Relationship of MPO and CIT-HIST-H3 with sex, DIC, and 7-day mortality among the studied sepsis patients.

Parameters		MPO (ng/ml)	Test value	P-value	CIT-HIST-H3 (ng/ml)	P-value
		Median (IQR)			Median (IQR)	
Sex	Male	73.7 (61.0–85.6)	– 0.518•	0.604	81.3 (66.6–175.4)	0.541•
	Female	75.0 (62.9–83.4)			112.3 (64.0–245.2)	
DIC occurrence	Positive	92.5 (56.8–110.9)	– 2.168•	0.030	257.8 (141.4–306.3)	0.000 •
	Negative	72.6 (62.1–80.4)			85.4 (60.0–135.1)	
7-day mortality	Alive	70.4 (58.5–79.0)	– 5.003•	0.000	92.2 (60.0–141.4)	0.000 •
	Died	109.0 (91.3–117.5)			269.9 (194.2–298.1)	

P-value > 0.05: Non-significant; P-value < 0.05: Significant; P-value < 0.01: Highly significant •: Mann-Whitney test.

**Table 8:** Correlation of MPO and CIT-HIST-H3 with other parameters studied among the sepsis patients.

Parameters	MPO (ng/ml)		CIT-HIST-H3 (ng/ml)	
	*r*	*P*-value	*r*	*P*-value
MPO (ng/ml)	–	–	0.244*	0.044
CIT-HIST-H3 (ng/ml)	0.244*	**0.044 s**	**–**	**–**
Age (years)	0.334**	**0.005**	0.108	0.376
HB g/dL	– 0.032	0.795	– 0.150	0.218
WBCs (x10^3^/ul)	– 0.053	0.663	0.066	0.587
PNL (x10^3^/ul)	0.128	0.293	0.251*	**0.037**
LYMP (x10^3^/ul)	– 0.231	0.056	– 0.371**	**0.002**
N/L ratio	0.258*	**0.032**	0.441**	**0.000**
PLTs (x10^3^/ul)	– 0.179	0.140	– 0.334**	**0.005**
PT (sec)	0.207	0.087	0.246*	**0.042**
INR	0.160	0.190	0.326**	**0.006**
PTT (sec)	0.150	0.234	0.249*	**0.045**
FDPs (< 5 Ug/ml)	0.195	0.108	0.233	0.054
D dimer (0-0.55 Ug/ml)	0.211	0.134	0.260	0.062
Fibrinogen (2–4 g/L)	-0.258*	**0.035**	– 0.294*	**0.016**
Procalcitonin (up to 0.05 ng/ml)	– 0.188	0.259	-0.104	0.534
CRP (< 5 mg/L)	– 0.115	0.410	-0.172	0.212
Creatinine mg/dl	0.214*	**0.014**	0.162	0.064
Total Bilirubin mg/dl	0.188*	**0.027**	0.124	0.149
PAI (N = 5-20ng/ml)	0.501**	**0.000**	0.249*	**0.039**
SOFA score	– 0.017	0.887	– 0.077	0.530
JAAM2 DIC score ≥ 3	0.293*	**0.015**	0.432**	**0.000**

P-value > 0.05: Non-significant; *P-value < 0.05: Significant; **P-value < 0.01: Highly significant. Spearman correlation coefficient.

### Evaluation of the differential and predictive performance of MPO and CIT-HIST-H3 in the different studied groups

#### MPO and CIT-HIST-H3 levels to differentiate between the patients and the control group (Table 9, Fig. 3)

**Table 9:** Receiver operating characteristics of MPO and CIT-HIST-H3 levels in distinguishing between patients and the control group.

Variable	Cut-off point	AUC	Sensitivity	Specificity	+PV	-PV
MPO (ng/ml)	> 3.7	0.999	98.55	100.00	100.0	95.2
CIT-HIST-H3 (ng/ml)	> 3.6	0.991	94.20	100.00	100.0	83.3

**Fig. 3:**
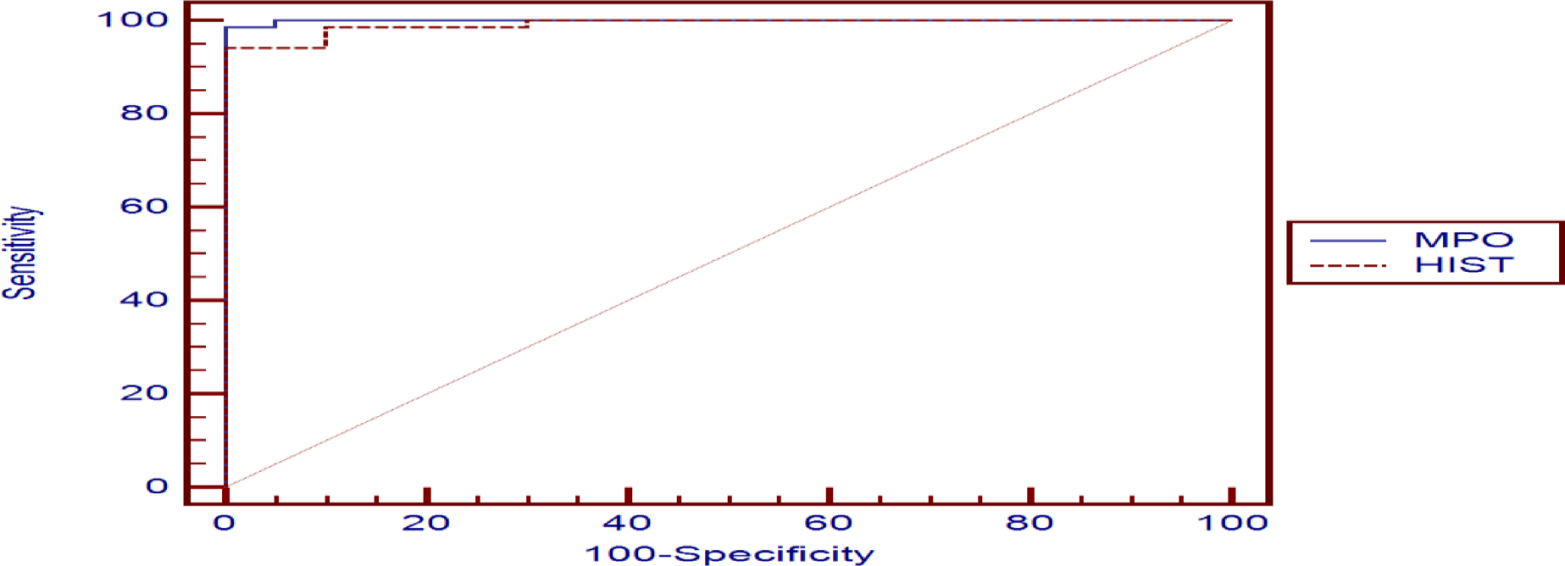
Receiver operating characteristic curve to differentiate between the sepsis patients and the healthy control group.

#### MPO and CIT-HIST-H3 levels to differentiate between positive-DIC and negative-DIC sepsis patients’ subgroups (Table 10, Fig. 4)

CIT-HIST-H3 showed higher sensitivity for DIC detection, while MPO exhibited greater specificity for DIC detection. Overall, their diagnostic performance was similar, with no significant difference as indicated by the p-value > 0.05.

**Table 10:** Receiver operating characteristics of MPO and CIT-HIST-H3 level to differentiate positive-DIC and negative-DIC sepsis patients’ subgroups:

Variable	Cut-off point	AUC	Sensitivity	Specificity	+PV	-PV	*P*-value (MPO vs. HIST)
MPO (ng/ml)	> 84.9	0.684	60.00	87.04	56.2	**88.7**	0.206
CIT-HIST-H3 (ng/ml)	> 126.4	0.826	86.67	74.07	48.1	**95.2**	

P-value > 0.05: Non-significant; *P-value < 0.05: Significant; **P-value < 0.01: Highly significant.

**Fig. 4:**
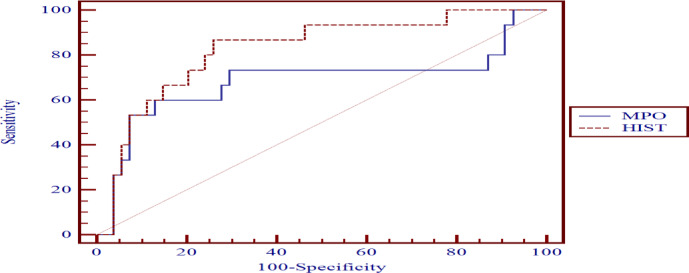
Receiver operating characteristics curve to differentiate positive-DIC and negative-DIC sepsis patients’ subgroups.

#### MPO and CIT-HIST-H3 levels to predict the 7-day mortality in sepsis patients (irrespective of the presence of DIC) (Table 11, Fig. 5)

MPO exhibited greater sensitivity and specificity for mortality prediction compared to CIT-HIST-H3, as indicated by the p-value < 0.05.

**Table 11:** Receiver operating characteristics of MPO and CIT-HIST-H3 levels for distinguishing 7-day deceased from 7-day surviving sepsis patients’ subgroup (irrespective of the presence of DIC).

Variable	Cut-off point	AUC	Sensitivity	Specificity	+PV	-PV	*P*-value (MPO vs. HIST)
MPO (ng/ml)	> 84.9	0.962	91.67	91.23	68.7	98.1	**0.027**
CIT-HIST-H3 (ng/ml)	**> 191.9**	0.848	83.33	85.96	55.6	96.1	

P-value > 0.05: Non-significant; *P-value < 0.05: Significant; **P-value < 0.01: Highly significant.

**Fig. 5:**
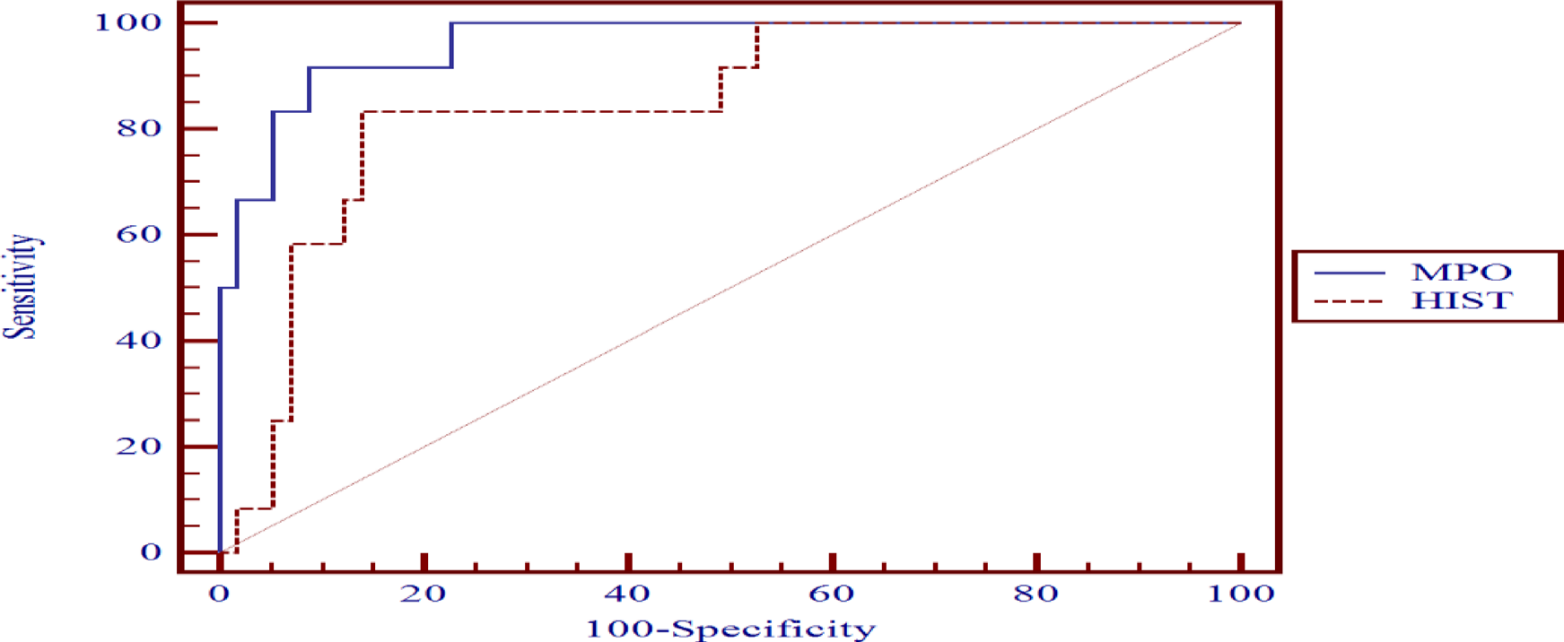
Receiver operating characteristic curve to distinguish between 7-day Dead and 7-day Alive Sepsis patient subgroups (irrespective of the presence of DIC).

## Discussion

In Critical care units, such as the ICU, the rates of morbidity and mortality among individuals suffering from sepsis or shock are alarmingly high. These patients often exhibit symptoms of systemic inflammatory response syndrome^14–16^. A crucial element in the onset of sepsis and the systemic inflammatory response is the activation of neutrophils, leading to the generation of NETs that enhance neutrophils’ capacity to fight infections^17,18^. If not adequately regulated, the formation of NETs can initiate a severe inflammatory response and lead to a procoagulant condition, potentially resulting in harmful blood clots or thrombosis^19–21^.

Considering strong evidence for the involvement of NETs—particularly CIT-HIST-H3 and MPO—in sepsis, our research aims to investigate their potential as diagnostic, prognostic, and therapeutic indicators in patients with sepsis in Critical care units.

### Demonstrating the intricate process of NETs formation in sepsis patients

Our research focused on measuring MPO and CIT-HIST-H3 levels across various participant groups, yielding insights into their roles in sepsis development. We observed a significant increase in both markers in sepsis patients compared to healthy controls. Importantly, we established cutoff values for MPO (> 3.7 ng/ml) and CIT-HIST-H3 (> 3.6 ng/ml) that effectively differentiate between the activation of NETs formation in sepsis patients and healthy individuals. Our study also highlighted a notable association between elevated levels of MPO and CIT-HIST-H3 in sepsis patients and several indicators, including high N/L ratios, increased JAAM scores used to diagnose DIC, and low serum fibrinogen levels. These findings provide a clear understanding of how these markers relate to the early, intense inflammatory response and coagulopathy commonly observed in sepsis, reflecting the physiological complexities these patients experience.

The role of neutrophil activation in sepsis—particularly the release of NETs that stimulate the production of ROS and MPO—has been emphasized in numerous studies^7,10,22^. Focusing on MPO, it is a pivotal enzyme in neutrophils that plays a crucial role in the inflammatory process by aiding in the production of ROS, which are essential for combating invading pathogens^23,24^. Additionally, HIST released from NETs has procoagulant properties, which can activate platelets, disrupt anticoagulant mechanisms, such as the protein C pathway, and inhibit the anticoagulant protein Antithrombin (AT)^10^.

Furthermore, a recent study by Zaiema et al. (2024) explored threshold levels of MPO and HIST for predicting the activation of NETs formation in patients with APLS and a reactive group (post-surgery, infection, or other inflammatory conditions), in contrast to healthy individuals. For MPO, a cutoff value of > 2.09 ng/mL was established as the most effective predictor of active NETs formation in APLS patients, with a cutoff of > 3.62 ng/mL identified for the reactive group. HIST H3 showed a cutoff of > 1.45 ng/mL as the most accurate predictor of active NETs formation in both APLS patients and their corresponding reactive group^25^.

It is essential to note that NETs are more problematic in sepsis than in autoimmune diseases. This severity arises from excessive, uncontrolled NETs formation, combined with impaired clearance processes. As a result, this can lead to widespread immunothrombosis and direct damage to multiple organs. In contrast, while NETs also play a significant role in autoimmune diseases, the extent of damage tends to be more localized. These situations usually involve a slower, chronic inflammatory process driven by specific autoantigens and a continuous cycle of autoantibody production. These observations indicate that establishing specific thresholds for NET activation may be crucial. Such cutoffs could facilitate the timely initiation of anti-NETosis therapies, thereby helping alleviate the adverse effects of NET formation across various medical conditions^26–28^. 

### Exploring the role of NETs formation in sepsis-induced coagulopathy

In our investigation, we conducted a detailed analysis of sepsis patients, specifically comparing those who developed DIC with those who did not. Our findings revealed several significant differences between these two groups. Notably, we observed a considerable increase in CIT-HIST-H3 and MPO levels in patients with DIC, with CIT-HIST-H3 showing a particularly marked rise. We also found that CIT-HIST-H3 was significantly associated with progressive thrombocytopenia and various abnormalities in coagulation markers (prolonged PT, PTT, and INR), as well as an elevated N/L ratio characterized by higher PNL and a lower lymphocyte count. The latter markers indicate an early inflammatory response that increases cortisol and catecholamine levels, aiming to restore immune balance, possibly through lymphocyte apoptosis^28–30^. These correlations were not seen with MPO. Additionally, the association between CIT-HIST-H3 and the elevated JAAM2 score—used to diagnose DIC—was notably stronger than that with MPO, emphasizing the critical role of HIST-H3 in sepsis.

To further assess the diagnostic potential of these biomarkers, we examined the ability of MPO and CIT-HIST-H3 to identify sepsis-related DIC in our patient group. Both markers showed similar moderate effectiveness, with MPO providing higher specificity at cutoffs > 84.9 ng/mL and CIT-HIST-H3 offering higher sensitivity at cutoffs > 126.4 ng/mL. The greater sensitivity of CIT-HIST-H3 is largely attributable to its prior association with coagulopathy and DIC. Overall, our study highlights the complex role of NETs formation, suggesting that higher levels of CIT-HIST-H3 have a more detrimental effect on sepsis-induced inflammation and coagulopathy (immunothrombosis) than MPO, opening avenues for future research and potential treatments.

The primary research on NETs formation in sepsis was carried out by Brinkmann et al. (2004) and Massberg et al. (2010), highlighting that NETs, as components of the innate immune response, help prevent the spread of microorganisms and ensure a high local concentration of antimicrobial substances to degrade virulence factors and kill bacteria^16,17^. A pilot investigation conducted by Delabranche et al. (2017) and Stiel et al. (2019) was the first to demonstrate visualization of circulating NETs by cell fluorescence in patients with septic shock-induced DIC, but not in those with septic conditions without DIC. They presented, for the first time, direct evidence of circulating NETs in the peripheral blood of individuals with septic shock-induced DIC, using immunofluorescence techniques. These NETs resemble the characteristics of NETs seen after normal PMNs are stimulated in vitro with ionomycin^18,19^.

Numerous recent reviews have emphasized the molecular mechanisms and overall impact of circulating histones in driving sepsis-induced coagulopathy and the progression of DIC in patients^8–10^. As highlighted in recent research, circulating histones, as they undergo post-translational modifications such as citrullination, contribute to DIC by accelerating fibrin polymerization, stabilizing clots against fibrinolysis, and directly binding to fibrin. Modified histones also cause endothelial injury and trigger the release of ultra-large von Willebrand factor multimers, thereby activating the tissue factor pathway^31,32,35^. Furthermore, histone-DNA complexes enhance coagulation by activating Factor XII-mediated pathways and oppose natural anticoagulant mechanisms, thereby directly promoting prothrombin autoactivation and exacerbating coagulopathy in sepsis^4,8–10^.

Unlike our findings, a prospective observational study by Sidana et al. (2022) indicated that HIST-H3 levels were not significantly higher in paediatric sepsis patients compared to controls, regardless of the severity of their conditions or clinical outcomes among children hospitalized with severe sepsis in a pediatric intensive care unit (PICU)^36^. This distinction underscores a potential limitation of HIST-H3 testing as a diagnostic biomarker in specific populations, like children with severe sepsis. Additionally, differences in the specificity of the HIST-H3 testing kit used in their study, compared with the one employed in our research, might account for this discrepancy.

### Exploring the role of NETs formation in sepsis-induced short-term mortality

Our goal was to enhance our understanding of how NETs influence short-term mortality in patients with sepsis. In our study, we identified key differences between patients who died within 7 days and those who survived, regardless of their DIC status. Notably, non-survivors tended to be older and showed higher rates of DIC and elevated PAI-1 levels. Additionally, these patients had higher levels of MPO and CIT-HIST-H3 than survivors. Interestingly, among patients with Positive-DIC, those who died within 7 days had significantly higher MPO levels, while CIT-HIST-H3 levels did not differ from those of Positive-DIC survivors.

We also found a strong correlation between higher MPO levels and older patients with sepsis, as well as elevated bilirubin and creatinine levels. These correlations were absent between those parameters and CIT-HIST-H3. Furthermore, the relationship between MPO and PAI-1—a key regulator of fibrinolysis known to be elevated in sepsis and a strong predictor of disease severity and organ damage^37^—was significantly stronger than that with CIT-HIST-H3. This prompted us to evaluate MPO’s predictive value for 7-day mortality in our cohort, revealing it to be a strong predictor of sepsis-related death, with a cutoff of 84.9 ng/mL, sensitivity of 91.67%, and specificity of 91.23%. Overall, our findings suggest that MPO has considerable potential as a prognostic marker for short-term mortality and correlates with markers of end-organ damage in sepsis, independent of DIC status. This underscores its potential as a critical clinical tool for the timely management of sepsis patients.

A pivotal study by Carr et al. (2020) examined plasma MPO levels in critically ill ICU patients and found significantly higher MPO concentrations in patients with septic shock compared with those without (302 ng/mL versus 156 ng/mL, respectively). Moreover, non-survivors had a mean MPO level of 416 ng/mL, starkly contrasting with the 140 ng/mL mean in survivors^24^. They also found that MPO is associated with markers of tissue injury and systemic organ failure in septic patients, such as higher APACHE III scores—a prognostic system used to predict hospital mortality in critically ill adult patients—suggesting MPO as a promising marker for enhancing mortality predictions^24^. We hypothesize that the higher MPO levels noted in Carr et al.‘s (2020) study among non-surviving sepsis patients may be attributable to differences in study design and the specific application of MPO assessments. Our research focused on a consecutive follow-up over seven days post-MPO assay as a predictor tool, specifically evaluating short-term mortality rates in sepsis patients.

Like our observations, Bonaventura et al. (2020) found that MPO’s ability to predict mortality in sepsis-induced coagulopathy decreased after the seventh day. This shows that MPO is a useful short-term marker but has limited value as a long-term predictor, underscoring the importance of early assessments^23^. Conversely, PAI-1 has been studied alongside various prognostic markers, including progressive thrombocytopenia, NLR, and SOFA scores, which are significant independent predictors of 28-day mortality in severe sepsis and sepsis-induced DIC^38–41^. Our research, focused specifically on predicting early mortality within the first 7 days, distinguishes itself from these earlier studies.

Furthermore, many studies have associated elevated MPO levels with high-risk factors such as metabolic syndrome, including increased body mass index and dyslipidemia, as well as stroke, diabetes, thromboembolism, and cardiovascular diseases. These are all signs of a significantly increased risk for future cardiovascular events^44–46^. Additionally, several clinical trials are currently investigating the therapeutic potential of targeting anti-MPO in various conditions, including sepsis, systemic lupus erythematosus, rheumatoid arthritis, small-vessel vasculitis, inflammatory bowel disease, cancer, and glomerulonephritis^7,13,31,47,48^.

Ultimately, factors that could cause variations in findings regarding MPO and HIST-H3 across studies include differences in disease severity and underlying mechanisms, as well as changes in patient demographics, sample sizes, timing, and the methods used to measure MPO and HIST-H3 levels^23,42^. Nevertheless, the current evaluation of MPO and HIST-H3 is hindered by lengthy turnaround times, which often exceed 3 days. This delay underscores the urgent need for a more efficient and reliable approach to harness the clinical potential of this vital biomarker.

## Conclusion

Our findings reveal a crucial link between NETs markers and the complex interactions of stress, inflammation, and coagulopathy inherent in septic conditions, as well as their impact on short-term mortality. Notably, histones emerged as a key factor in sepsis-induced immunothrombosis and DIC. MPO showed a strong correlation with short-term mortality and adverse prognostic markers such as older age, signs of organ dysfunction, and PAI. These insights highlight the significant relationship between NETs formation and clinical outcomes in sepsis patients.

Furthermore, an alarming, combined increase in MPO and Histones—thresholds of > 84.9 ng/ml for MPO and > 126.4 ng/ml for Histones—could serve as an urgent warning to implement NETs inhibitors in sepsis management. Using this approach, we anticipate a significant reduction in thrombotic events and mortality, ultimately improving care and outcomes for individuals affected by this severe illness.

Additional clinical research and trials are needed to confirm the clinical significance of our proposed cutoff values and to establish a universal reference range for NETs markers across diverse age groups, genders, and health conditions. Moreover, it is essential to deepen our understanding of the balance between the natural immune role of NETs and the possible effects of NETs inhibitors in treating sepsis.

## Data Availability

The datasets used and/or analysed during the current study are available from the corresponding author on reasonable request on [https://drive.google.com/file/d/1ogyQ1rS8ed\_EFjSi1SYYjKRG5 × 0CYnnP/view? usp=sharing](https:/drive.google.com/file/d/1ogyQ1rS8ed\_EFjSi1SYYjKRG5 × 0CYnnP/view? usp=sharing) . Further Data sharing is allowed for this article from the corresponding author upon reasonable request, as datasets were generated and analyzed during the current study.

## Abbreviations

NETs: Neutrophil extracellular traps
MPO: Myeloperoxidase
HIST: Histones
Cit-HIST-H3: Human citrullinated histone H3
ROS: Reactive oxygen species
ELANE: Neutrophil elastase
AT: Antithrombin
SIC: Sepsis-induced coagulopathy
DIC: Disseminated intravascular coagulation
SIRS: Systemic inflammatory response syndrome
SOFA: Sequential organ failure assessment
JAAM-2: Japanese association for acute medicine modified score
NLR: Neutrophil-to-lymphocyte ratio
FDP: Fibrin degradation products
INR: International normalized ratio
PAI-1: Plasminogen activator inhibitor-1
TTP: Thrombotic thrombocytopenic purpura
HUS: Hemolytic uremic syndrome
PCT: Pro-calcitonin
CRP: C-reactive protein
ELISA: Enzyme-linked immunosorbent assay
PLT: Platelets
Hb: Hemoglobin
APLS: Antiphospholipid syndrome
